# Characterization of *Plasmodium relictum*, a cosmopolitan agent of avian malaria

**DOI:** 10.1186/s12936-018-2325-2

**Published:** 2018-05-02

**Authors:** Gediminas Valkiūnas, Mikas Ilgūnas, Dovilė Bukauskaitė, Karin Fragner, Herbert Weissenböck, Carter T. Atkinson, Tatjana A. Iezhova

**Affiliations:** 1 Nature Research Centre, Akademijos 2, LT-08412 Vilnius, Lithuania; 2 Institute of Pathology and Forensic Veterinary Medicine, University of Veterinary Medicine, Vienna, 1210 Vienna, Austria; 3 U.S. Geological Survey, Pacific Island Ecosystems Research Center, Hawaii National Park, HI 96718 USA

**Keywords:** *Plasmodium relictum*, Birds, Morphological and molecular characterization, Review

## Abstract

**Background:**

Microscopic research has shown that *Plasmodium relictum* is the most common agent of avian malaria. Recent molecular studies confirmed this conclusion and identified several mtDNA lineages, suggesting the existence of significant intra-species genetic variation or cryptic speciation. Most identified lineages have a broad range of hosts and geographical distribution. Here, a rare new lineage of *P. relictum* was reported and information about biological characters of different lineages of this pathogen was reviewed, suggesting issues for future research.

**Methods:**

The new lineage pPHCOL01 was detected in Common chiffchaff *Phylloscopus collybita,* and the parasite was passaged in domestic canaries *Serinus canaria*. Organs of infected birds were examined using histology and chromogenic in situ hybridization methods. *Culex quinquefasciatus* mosquitoes, Zebra finch *Taeniopygia guttata,* Budgerigar *Melopsittacus undulatus* and European goldfinch *Carduelis carduelis* were exposed experimentally. Both Bayesian and Maximum Likelihood analyses identified the same phylogenetic relationships among different, closely-related lineages pSGS1, pGRW4, pGRW11, pLZFUS01, pPHCOL01 of *P. relictum.* Morphology of their blood stages was compared using fixed and stained blood smears, and biological properties of these parasites were reviewed.

**Results:**

Common canary and European goldfinch were susceptible to the parasite pPHCOL01, and had markedly variable individual prepatent periods and light transient parasitaemia. Exo-erythrocytic and sporogonic stages were not seen. The Zebra finch and Budgerigar were resistant. Neither blood stages nor vector stages of all examined *P. relictum* lineages can be distinguished morphologically.

**Conclusion:**

Within the huge spectrum of vertebrate hosts, mosquito vectors, and ecological conditions, different lineages of *P. relictum* exhibit indistinguishable, markedly variable morphological forms. Parasites of same lineages often develop differently in different bird species. Even more, the variation of biological properties (parasitaemia dynamics, blood pathology, prepatent period) in different isolates of the same lineage might be greater than the variation in different lineages during development in the same species of birds, indicating negligible taxonomic value of such features. Available lineage information is excellent for parasite diagnostics, but is limited in predictions about relationships in certain host-parasite associations. A combination of experiments, field observations, microscopic and molecular diagnostics is essential for understanding the role of different *P. relictum* lineages in bird health.

**Electronic supplementary material:**

The online version of this article (10.1186/s12936-018-2325-2) contains supplementary material, which is available to authorized users.

## Background

*Plasmodium relictum* is an invasive blood parasite, which causes malaria in many species of birds from all over the word [1–4]. Naive birds often experience severe disease and even mortality during malaria infection [5, 6], but some bird species and their populations appear to be relatively resistant and can tolerate this infection [7–11]. This parasite was the first recognized and described agent of avian malaria [12], likely due to its high prevalence in a wide range of different avian hosts and because morphological characteristics of its mature blood stages are so distinctive in blood films. Mature stages typically possess prominent nuclei and cytoplasm, numerous pigment granules and markedly influence the position of the nuclei of their host erythrocytes, causing lateral shifts in their position. Numerous synonymous names of this organism exist [7, 13]. These names were suggested for distinguishing the morphologically similar or even identical blood stages, which were reported in different avian hosts and/or different geographical areas [13–15]. Microscopic examination of blood films, the main avian malaria diagnostic tool used in the 20th Century, has identified *P. relictum* as the most common agent of avian malaria with reports from over 300 species of birds belonging to 11 orders from all over the world [1, 7, 16, 17]. Recent molecular studies have supported this conclusion and uncovered significant genetic diversity among different isolates of *P. relictum*, suggesting existence of intra-species genetic variation or even cryptic speciation [2, 18–22].

Partial sequences of mitochondrial cytochrome *b* gene (*cytb*) have been successfully used in distinguishing different lineages of avian malaria parasites, and they are excellent molecular markers for disease diagnostics [2, 4, 19, 23–25]. Over 100 closely related *cytb* lineages of avian *Plasmodium* were deposited in GenBank and MalAvi database (http://mbio-serv2.mbioekol.lu.se/Malavi) and many of them may belong to *P. relictum*. However, few of these molecular lineages are supported by microscopic examination of well-fixed and stained blood smears and the small genetic difference in *cytb* sequences alone cannot be considered as final proof that closely related lineages belong to the same morpho-species. For example, some morphologically distinct haemosporidian species differ in their partial *cytb* sequences just by a few nucleotide bases [26, 27]. Currently, only four lineages (pSGS1, pGRW4, pGRW11, pLZFUS01) have been linked to *P. relictum* based on morphological characters of their blood stages, and these data are helpful for distinguishing this infection in blood films [28–30].

During the past 15 years, much data have been collected about host, geographical distribution, vectors, virulence, and other biological characters of *P. relictum* based on *cytb* lineages [2, 3, 8, 19, 30–34]. This provides opportunities to examine patterns in the biology and pathology of avian *Plasmodium* infection at the level of these specific lineages. This study characterizes a new *cytb* lineage of *P. relictum* (pPHCOL01), makes comprehensive comparisons of morphological characters of blood stages of all known lineages of this parasite, and reviews their biological features to help identify some new directions for future avian malaria research.

## Methods

### Collection of blood and tissue samples

Fieldwork was carried out at the Ventės Ragas Ornithological Station, Lithuania between 4 and 18 May, 2017. Twenty-three Common chiffchaffs *Phylloscopus collybita* were caught with mist nets and large stationary traps. The blood was taken by puncturing the brachial vein. Three blood films were prepared immediately after withdrawal of the blood, air-dried using a battery-operated fan, fixed in absolute methanol and stained with Giemsa. About 30 μl of whole blood was taken in heparinized microcapillaries and stored in SET buffer (0.05 M Tris, 0.15 M NaCl, 0.5 M EDTA, pH 8.0) at ambient temperature while in the field and then maintained at − 20 °C in the laboratory.

To detect and isolate the *Plasmodium* parasite strain, the blood films from each captured bird were quickly examined microscopically in the field, as previously described [35]. One naturally infected Common chiffchaff was detected, with parasitaemia of 0.1%. Ten blood films were prepared for microscopic examination from this bird. Additionally, blood was also collected in heparinized microcapillaries and used to expose two uninfected domestic Common canaries *Serinus canaria* forma *domestica* by sub-inoculation into their pectoral muscle of about 250 μl of a freshly prepared mixture of infected blood, 3.7% sodium citrate (anticoagulant) and 0.9% saline (4:1:5) [36].

Parasitaemia developed in both exposed canaries, and blood of these birds was passaged as described above in three additional canaries. Two Zebra finches *Taeniopygia guttate,* one Budgerigar *Melopsittacus undulatus* and two European goldfinches *Carduelis carduelis* were also exposed, as described above. Six uninfected canaries were used as controls. Blood of all control and experimental birds was tested by microscopic examination and PCR-based methods (see description below) twice before the experiment to ensure that they were uninfected with malaria parasites.

Two canaries were observed for 57 and 94 days post exposure (dpe) and then euthanized for histology and chromogenic in situ hybridization research. Two European goldfinches were observed for 127 dpe. All remaining birds were observed for 131 dpe. Post-exposure blood samples were taken for microscopic examination and PCR-based testing as described above once every 4 days during the first post-exposure month, once every week during the second month and once every 1–2 weeks during the remaining experiment time. All experimental and control birds were kept indoors in a vector-free room under natural light–dark photoperiod. They were fed a standard diet for seed-eating bird species.

Control birds were maintained in the laboratory for further research. The donor Common chiffchaff and infected experimental birds were euthanized, and pieces of organs (brain, liver, lungs, heart, kidneys, spleen) and pieces of pectoral muscles were fixed in 10% neutral formalin, embedded in paraffin and processed using traditional histologic methods [7]. Histological sections of 4 μm were obtained, stained with haematoxylin–eosin, mounted in BioMount (BioGnost, Croatia) and examined microscopically. One smear of bone marrow was prepared from the tibia bone of each bird, air dried, fixed with absolute methanol and stained with Giemsa.

### Experimental infection and investigation of *Culex quinquefasciatus* mosquitoes

Laboratory-reared *Culex quinquefasciatus* mosquitoes were maintained and exposed to canaries infected with the isolated *Plasmodium* sp., as previously described [32]. Briefly, insects were kept in cages (45 × 45 × 45 cm) under 65–70% relative humidity, 16/8 h light/dark photoperiod and 26 ± 1 °C. One experimentally infected canary was used as the donor of parasites for infecting mosquitoes. Eleven female mosquitoes took blood meals on this canary. Parasitaemia was 0.02% with few visible mature gametocytes. Preparations of midgut contents (6 insects were dissected 24–48 h post exposure), one preparation of midgut wall (one insect on 12 dpe) and 4 preparations of salivary glands (on 15, 16 and 18 dpe) were prepared and examined, as previously described [32].

### Microscopic examination

Detailed microscopic analysis was carried out with various Olympus light microscopes equipped with Olympus digital cameras and imaging software. Preparations of blood stages of the lineages pSGS1, pGRW11, pGRW4, and pLZFUS01 were from collections of voucher specimens which have been deposited at P. B. Šivickis Laboratory of Parasitology, Nature Research Centre Vilnius. These were blood films from canaries whose were exposed experimentally to the parasite lineages pSGS1 (parasitaemia varied between 0.6 and 1.8%, preparation accession number 48979–48981 NS), pGRW11 (1.1–6%, 48982–48984 NS), pGRW4 (0.2–2.1%, 48985–48987 NS), and the lineage pLZFUS01 (0.5%, 48694–48696 NS) from the blood of a naturally infected Red-backed shrike *Lanius collurio* (for exposure description see [30]). Additionally, preparations with the Hawaiian isolate of *P. relictum* (pGRW4) were used. These were (1) 12 blood films from two individual canaries that were exposed experimentally by inoculation of infected blood (parasitaemia varies between 0.6 and 10%, accession nos. 48988–48999 NS) (for infection details see [5]), (2) 11 blood films from one naturally infected Apapane *Himatione sanguinea* (parasitaemia 22%, accession nos. 49000–49010 NS).

Blood films from each infected bird were examined and the observed blood stages were morphologically compared by skilled parasitologists of avian malaria parasites at the P. B. Šivickis Laboratory of Parasitology. At least 100 fields were studied at high magnification (1000×) in each preparation. Intensity of parasitaemia was estimated as a percentage by actual counting of the number of parasites per 10,000 erythrocytes. The morphometric features studied (Table 1) were those defined in [7]. The analyses were carried out using the ‘Statistica 7’ package as previously described [7].

**Table 1:** Morphometry of blood stages and host cells of *Plasmodium (Haemamoeba) relictum* (pPHCOL01) from the blood of Common chiffchaff *Phylloscopus collybita* (n = 21)

Feature	Measurements (μm)^a^
Uninfected erythrocyte	
Length	10.5–11.9 (11.2 ± 0.4)
Width	5.2–6.4 (5.8 ± 0.3)
Area	46.9–55.6 (51.7 ± 2.7)
Uninfected erythrocyte nucleus	
Length	5.2–6.1 (5.6 ± 0.3)
Width	1.8–2.3 (2.0 ± 0.1)
Area	8.1–10.8 (9.7 ± 0.7)
Macrogametocyte	
Infected erythtocyte	
Length	8.3–12.2 (10.6 ± 1.1)
Width	6.2–8.1 (7.0 ± 0.6)
Area	43.9–70.8 (58.3 ± 6.8)
Infected erythrocyte nucleus	
Length	4.5–6.8 (5.4 ± 0.5)
Width	2.1–3.4 (2.6 ± 0.3)
Area	9.2–15.2 (11.4 ± 1.3)
Gametocyte	
Length	5.9–8.0 (7.1 ± 0.5)
Width	3.2–4.1 (3.6 ± 0.3)
Area	15.5–22.7 (20.3 ± 1.9)
Gametocyte nucleus	
Length	2.1–3.1 (2.6 ± 0.3)
Width	1.4–2.5 (2.0 ± 0.3)
Area	2.6–5.3 (4.2 ± 0.6)
Pigment granules	8.0–17.0 (12.0 ± 2.3)
Microgametocyte	
Infected gametocyte	
Length	8.6–12.7 (10.7 ± 1.1)
Width	6.0–8.5 (7.1 ± 0.7)
Area	50.4–67.8 (59.1 ± 4.5)
Infected erythrocyte nucleus	
Length	5.0–6.3 (5.5 ± 0.4)
Width	1.8–3.3 (2.5 ± 0.4)
Area	9.4–14.8 (11.7 ± 1.4)
Gametocyte	
Length	6.1–10.0 (7.7 ± 0.9)
Width	3.5–5.4 (4.4 ± 0.5)
Area	18.9–29.4 (24.4 ± 2.7)
Gametocyte nucleus	
Length	3.0–5.0 (4.0 ± 0.5)
Width	2.4–3.8 (2.9 ± 0.4)
Area	6.4–12.8 (8.5 ± 1.7)
Pigment granules	9.0–16.0 (12.4 ± 1.8)
Meront	
Length	4.5–7.8 (5.8 ± 0.8)
Width	3.1–5.6 (4.4 ± 0.5)
Area	13.6–33.7 (19.5 ± 4.6)
Area of pigment granules	0.8–1.6 (1.2 ± 0.3)
No. of merozoites	10–22 (18.9 ± 3.8)

^a^Minimum and maximum values are provided, followed in parentheses by the arithmetic mean and standard deviation

### In situ hybridization

Chromogenic in situ hybridization (ISH) was applied to increase detectability of tissue stages of the parasites. Organs (the same as for histological examination) from one naturally infected Common chiffchaff and two experimentally infected canaries (57 and 94 dpe, respectively) were tested using a previously described ISH protocol [37]. 3 μm paraffin-embedded tissue sections of all these organs were prepared. The sections were deparaffinized, subjected to proteolytic treatment with proteinase K (Roche, Basel, Switzerland) 6 μg/ml in Tris-buffered saline at 37 °C for 50 min. For hybridization, the slides with tissue sections were incubated overnight at 40 °C with hybridization mixture and a final probe concentration of 100 ng/ml. The used oligonucleotide probe (sequence: 5′-TTTAATAACTCGTTATATATATCAGTGTAGCAC-3′) was labelled with digoxigenin at the 3′ end (Eurofins MWG Operon, Ebersberg, Germany). This probe is specific to detect avian *Plasmodium* parasites [6, 37]. The digoxigenin-labelled hybrids were detected by incubating the slides with anti-digoxigenin-AP Fab fragments (Roche) (1:200) for 1 h at room temperature (RT). Visualization of the reaction was carried out using the colour substrates 5-bromo-4-chloro-3-indolyl phosphate (BCIP) and 4-nitro blue tetrazolium chloride (NBT) (Roche). A positive control (sections of a lung of a Eurasian blackbird *Turdus merula* naturally infected with *Plasmodium vaughani*, which was proven to be positive by previous ISH) was used to assure that the protocol worked. Preparations were examined microscopically by skilled parasitologists and pathologists; at least 50 fields of each preparation were studied at low magnification (400×), and then each preparation was examined for 10–15 min at high magnification (1000×).

### Molecular and phylogenetic analysis

Total DNA was extracted from blood samples using an ammonium-acetate precipitation protocol [38]. Polymerase chain reaction using the primer set HaemNFI/NR3 and Haem/R2 was performed in order to amplify a 479 bp sequence of the parasite’s *cytb* gene [18, 39]. The total volume of the reaction mix for each sample was 25 µl, which included 12.5 µl of DreamTaq Master Mix (Thermo Fisher Scientific, Lithuania), 8.5 µl nuclease-free water, 1 µl of each primer and ~ 50 ng of a total genomic DNA template (2 µl). Negative controls (nuclease-free water) were used after each seven samples to detect possible false amplifications, and one positive control (extracted parasite DNA from a blood sample, which was confirmed positive during previous PCR testing) was used to evaluate the success of PCR if none of the samples would have been amplified.

Temperatures for the PCR were as described in the original protocols. The success of the performed PCR was evaluated by running electrophoresis on a 2% agarose gel. Successfully amplified DNA was precipitated using 11 µl of 8 M NH 4Ac, 37 µl of 96% and 150 µl of 70% ethanol. After centrifugation, the supernatant was discarded, the samples were air-dried overnight, and then 16 µl of nuclease-free water was added on the precipitated DNA. Big Dye Terminator V3.1 Cycle Sequencing Kit and ABI PRISM™ 3100 capillary sequencing robot (Applied Biosystems, Foster City, California) were used for sequencing. Sequences were edited and aligned using BioEdit software [40]. Absence of double-base calling in sequence electropherograms was used as an indication of single infections [41]. Nucleotide BLAST (megablast algorithm) (http://blast.ncbi.nlm.nih.gov/Blast.cgi) was used to compare our amplified sequences with sequences deposited in the GenBank.

**Fig. 1:**
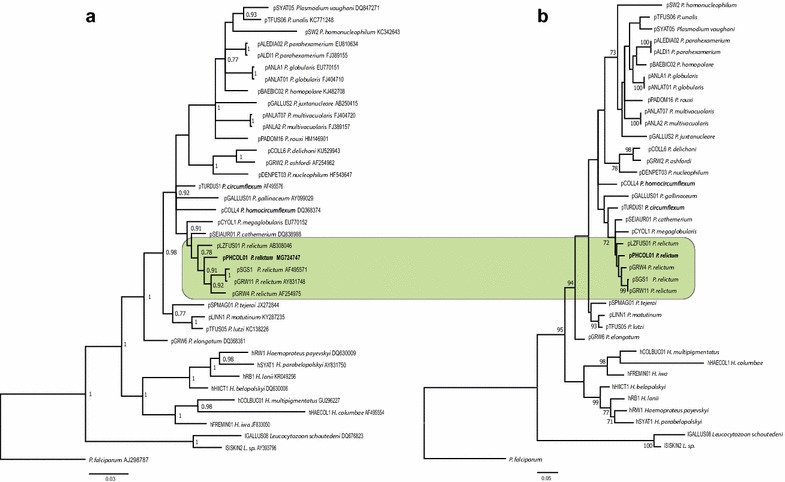
Bayesian (**a**) and Maximum Likelihood (**b**) phylogeny of 29 mitochondrial cytochrome *b* lineages of *Plasmodium* species, seven lineages of *Haemoproteus* spp. and two lineages of *Leucocytozoon* spp. One lineage of *Plasmodium falciparum* is used as an outgroup. Codes of the lineages (according to MalAvi database, http://mbio-serv2.mbioekol.lu.se/Malavi), parasite species names and GenBank accession numbers are provided on the trees. Posterior probabilities of > 0.7 (**a**) and bootstrap values of > 70 (**b**) are indicated near the nodes. The branch lengths are drawn proportionally to the amount of change (scale bars are shown). Green rectangle indicates the clade of closely related *Plasmodium relictum* lineages, which were analysed in this study. The new lineage pPHCOL01 is given bold

Molecular phylogenetic analysis was carried out using Bayesian and Maximum Likelihood algorithms. Sequences for the phylogenetic analysis were collected from GenBank and double-checked in MalAvi database [19]. *Plasmodium falciparum* was used as an outgroup. GenBank accession numbers and codes of the lineages are provided in the phylogenetic trees (Fig. 1). Bayesian phylogenetic tree (Fig. 1a) was constructed using MrBayes version 3.1 [42] software. The General Time Reversible Model (GTR) was used as suggested by the software MrModeltest 2.2 (https://github.com/nylander/MrModeltest2). Analysis was run for a total of 10 million generations with a sampling frequency of every 100 generations. Before the construction of the consensus tree, 25% of the initial trees were discarded as ‘burn in’ period. The tree was visualized using the software FigTree v1.4.3 (http://tree.bio.ed.ac.uk/software/figtree/). Maximum Likelihood tree (Fig. 1b) was constructed using the MEGA 7.0 [43] software; it was performed with 1,000 bootstrap replications using the GTR model and the same dataset as during the Bayesian analysis.

The new sequence of lineage pPHCOL01 was deposited in GenBank (accession MG724747). Genetic differences between different lineages of *P. relictum* were calculated using the Jukes–Cantor model of substitution, as implemented in the programme MEGA 7.0 [43].

## Results

### Parasite lineage identification and susceptibility of experimental birds

Single infections of *P. relictum* (*cytb* lineage pPHCOL01) was identified in the donor Common chiffchaff both by microscopic examination of blood films and PCR-based amplification and sequencing. All exposed canaries were susceptible and developed a single infection with the same malaria parasite, as determined both by microscopic examination of blood films and PCR-based testing. Parasitaemia developed in one exposed European goldfinch. Two Zebra finches, one Budgerigar and one European goldfinch were resistant. All control canaries remained non-infected during this study.

### Phylogenetic analysis

The reported lineage of *P. relictum* (pPHCOL01) was new. It clustered with other morphologically characterized lineages of *P. relictum* (pSGS1, pGRW4, pGRW11, pLZFUS01) in both phylogenetic analyses, supporting the close phylogenetic relationships among them (Fig. 1a, b). Genetic differences among five lineages of *P. relictum* varied between 0.2% (minimum, the lineages pSGS1 and pGRW11) and 3% (maximum, the lineages pSGS1 and pLZFUS01).

### Characterization of *Plasmodium (Haemamoeba) relictum* (pPHCOL01)

**Fig. 2:**
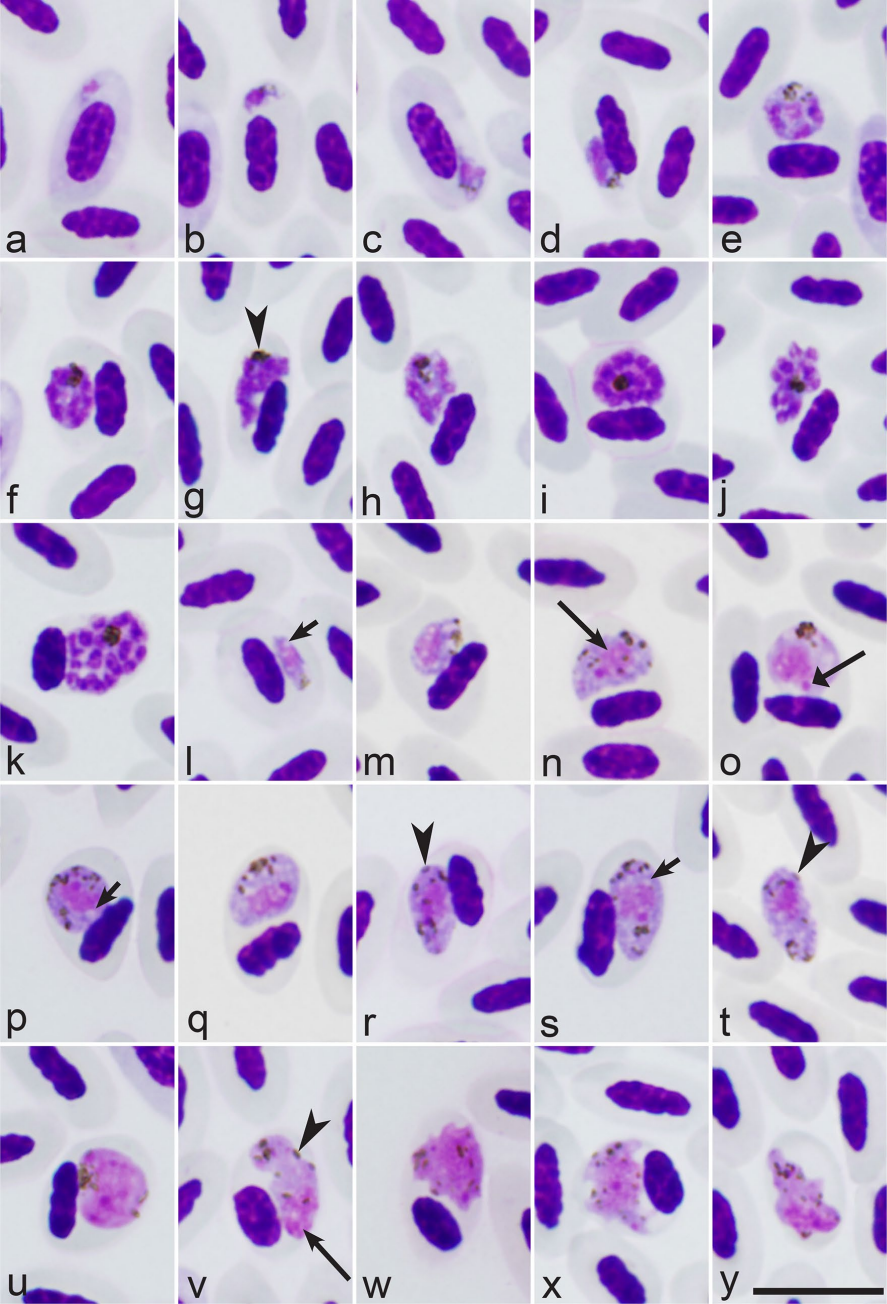
*Plasmodium relictum* (lineage pPHCOL01) from the blood of Common chiffchaff *Phylloscopus collybita*. **a**–**d**—trophozoites; **e**–**k**—erythrocytic meronts; **l**–**t**—macrogametocytes; **u**–**y**—microgametocytes. Long arrows—parasite nuclei. Short arrows—vacuoles. Arrowheads—pigment granules. Triangle wide arrow—nucleolus. Giemsa-stained thin blood films. Scale bar = 10 μm

See (Fig. 2, Table 1).

#### DNA sequence

Mitochondrial *cytb* lineage pPHCOL01 (new lineage, 479 bp, GenBank accession MG724747).

#### Avian hosts

Common chiffchaff *Phylloscopus collybita* is a natural host. Other natural avian hosts are unknown. Two Zebra finches and one Budgerigar that were exposed by sub-inoculation of infected blood were resistant. The most similar *Plasmodium* parasite lineages were reported only in sub-Saharan birds by Loiseau et al. [44] (the lineage PV40, accession HQ022817, 2 bp difference, avian host was not reported), Beadell et al. [2] (the lineage P27, accession DQ659568, 7 bp difference, the host is the Cameroon sunbird *Cyanomitra oritis)* and Lutz et al. [45] (the lineage P_AFR110, accession KM056570, 7 bp difference, the host is the Miombo tit *Parus griseiventris*).

#### Vectors

Remain unidentified. Sporogonic development was not observed in *Culex quinquefasciatus* mosquitoes.

#### Site of infection

Red blood cells; no other data.

#### Representative blood films

Voucher specimens (accession numbers 48965–48974 NS, *Phylloscopus collybita,* 7 May 2017, parasitaemia 0.1%, collected by D. Bukauskaitė, and 48975–48978 NS, *Serinus canaria*, 2–6 June, 2017, collected by M. Ilgūnas) were deposited in Nature Research Centre, Vilnius, Lithuania.

#### Prevalence

The overall prevalence was 1 of 23 (4.3%) in Common chiffchaff at the study site.

#### Parasitaemia and virulence

Canaries are susceptible, with long-lasting (up to 65 dpe), but light parasitaemia (< 0.01%) reported in the majority of exposed birds. One of two exposed European goldfinches developed very light (0.001%) and long-lasting (up to 127 dpe) parasitaemia. In all positive birds, parasitaemia was transient, i.e., it was not seen during all days of testing. In experimentally exposed birds, the maximum reported parasitaemia was 0.02%, and it was seen in one canary. The parasitaemia remained light or even declined into latency approximately 1–2 weeks after the first parasites seen in blood films in all positive birds, with a few parasites appearing in the circulation during entire observation time.

All blood stages (trophozoites, growing and mature meronts, growing and mature gametocytes) were reported in the peripheral circulation of naturally infected Common chiffchaff, experimentally exposed canaries and one goldfinch. This indicates asynchronous development in the blood. Mortality was not reported among exposed birds, and they appeared healthy. Clinical signs of disease were not observed during this study, and it is probable that susceptible inoculated birds can tolerate this infection.

The prepatent period varied markedly, with first parasites observed in the peripheral circulation 9, 14, 31, and 49 dpe in different canaries. Prepatent period was 11 dpe in one European goldfinch.

Morphology of blood stages of the new lineage parasites was the same in the Common chiffchaff and the experimentally exposed canaries and one goldfinch. Description of blood stages of this infection is given from preparations with parasitaemia of 0.1% in Common chiffchaff (Fig. 2).

#### Trophozoites

Figure 2a–d. Develop mainly in mature erythrocytes (Fig. 2b, d), but sometimes were also seen in polychromatic erythrocytes (Fig. 2a, c). Earliest trophozoites usually are of irregular form, often amoeboid in outline (Fig. 2a, b); they only slightly displace nuclei in infected erythrocytes laterally. Advanced trophozoites possess prominent nuclei and cytoplasm, but lack vacuoles (Fig. 2c, d); they were often attached to the host cell nuclei (Fig. 2d), which are slightly displaced. Pigment granules are roundish, small (< 0.5 µm), few, dark-brown, and usually grouped.

#### Erythrocytic meronts

Figure 2e–k. Develop in mature erythrocytes. Young growing meronts possess plentiful cytoplasm and large nuclei (Fig. 2e); size of the nuclei and amount of the cytoplasm markedly decrease as the parasites mature (compare Fig. 2e with Fig. 2f–h). Vacuoles are absent from both developing and mature meronts. Pigment granules are small (< 0.5 µm), dark-brown or black, usually grouped in young meronts (Fig. 2e), clumped and difficult to calculate in mature meronts (Fig. 2g–k). Mature meronts produce up to 22 merozoites (Table 1), which are usually arranged haphazardly (Fig. 2j, k). Growing and mature meronts markedly displace nuclei of infected erythrocytes (Fig. 2e–k) and sometimes enucleate the host cells. Meronts were uncommon in peripheral circulation.

#### Macrogametocytes

Figure 2l–t. Predominate in peripheral circulation; they develop in mature erythrocytes. Growing and mature gametocytes are markedly variable in form, with roundish (Fig. 2o, p), oval (Fig. 2m, r–t) and various irregular shapes (Fig. 2n) present. Numerous growing and mature gametocytes adhere to the nuclei of erythrocytes (Fig. 2m, o, p, r, s). Gametocytes adhering to the erythrocyte nuclei predominate, but the gametocytes, which do not touch nuclei of erythrocytes were also seen (Fig. 2n, q). Small vacuoles were reported in the cytoplasm occasionally (Fig. 2l, p, s). Parasite nucleus is prominent, of irregular shape; nucleolus is readily visible (Fig. 2o). Pigment granules are small (< 0.5 µm) or of medium size (0.5–1 µm), black or dark-brown, mainly roundish or oval (Fig. 2n, p, q), but elongate pigment granules were seen occasionally (Fig. 2r); pigment granules are scattered in the cytoplasm (Fig. 2n, r, t) or sometimes grouped (Fig. 2o, q). Gametocytes markedly deform the infected red blood cells and displace their nuclei toward one of poles of the host cells; they often enucleate the infected cells (Fig. 2t).

#### Microgametocytes

Figure 2u–y. General configuration and other features are as for macrogametocytes, with usual haemosporidian sexual dimorphic characters, which are the pale staining of the cytoplasm and the diffuse large nuclei. Irregular-shape mature gametocytes are common (Fig. 2w–y).

### Remarks

**Fig. 3:**
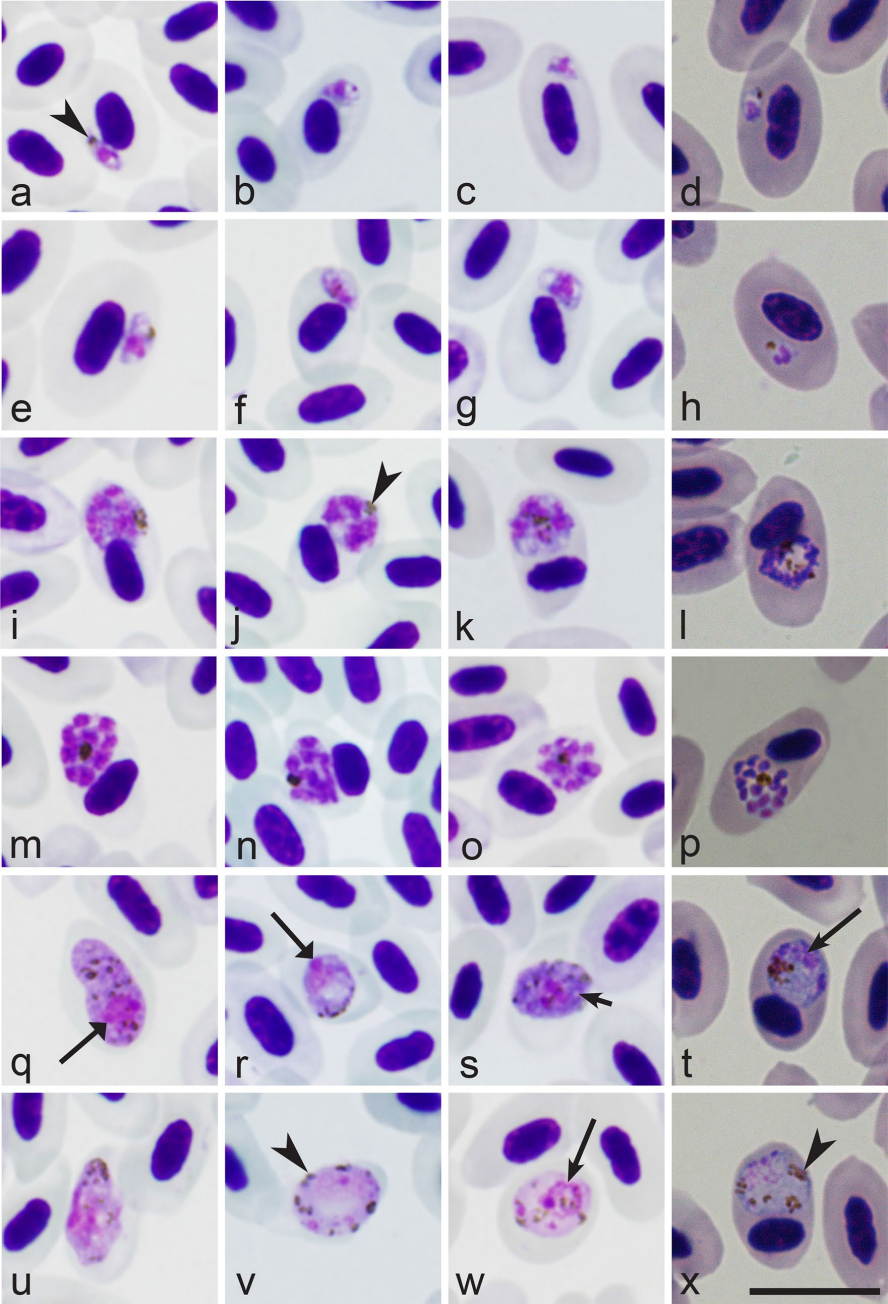
*Plasmodium relictum* lineages pSGS1 (**a**, **e**, **i**, **m**, **q**, **u**), pGRW4 (**b**, **f**, **j**, **n**, **r**, **v**), pGRW11 (**c**, **g**, **k**, **o**, **s**, **w**) and pLZFUS01 (**d**, **h**, **l**, **p**, **t**, **x**). **a**–**d**—early trophozoites, **e**–**h**—advanced trophozoites, **i**–**l**—developing meronts, **m**–**p**—mature meronts, **q**–**t**—macrogametocytes, **u**–**x**—microgametocytes. All parasites are from the blood of domestic canary *Serinus canaria*, except for the pLZFUS01 lineage parasites, which are from the blood of Red-backed shrike *Lanius collurio.* Symbols are the same as in Fig. 2

Examination of all blood films with the *P. relictum* lineages pSGS1, pGRW4, pGRW11, pLZFUS01 (Fig. 3) revealed the morphological identity of trophozoites, meronts and macro- and microgametocytes of these parasites in all infections that were examined. Number of merozoites in mature erythrocytic meronts of all parasite lineages and different isolates of the same lineage is markedly variable during development in the same and different species of avian hosts; it varied between 10 and 32 merozoites, but most often reported to be between 12 and 24 merozoites in all examined infections. These lineages of *P. relictum* cannot be distinguished based on this character. Additionally, the main morphological forms of blood stages reported in parasites of the new lineage pPHCOL01 (Fig. 2) were seen in blood films with single infection of all other lineage of *P. relictum* in the same and different species of avian hosts (Fig. 3). Variation in shape of each blood stage of *P. relictum* occurs, but all observed morphological forms of blood stages (Figs. 2a–y, 3a–x) were seen in parasites belonging to each examined parasite lineage. In other words, the morphological forms of all blood stages (trophozoites, growing and mature meronts, growing and mature gametocytes) in all examined *P. relictum* lineages were indistinguishable.

Interestingly, two different isolates of the lineage GRW4 (the Hawaiian strain and the strain isolated in Europe) produced indistinguishable trophozoites, meronts and gametocytes during development in canaries. Additionally, extensive microscopic examination showed that morphological and morphometric characters of blood stages of the widespread lineages pGRW4 and pSGS1 were variable during development in same and different avian hosts, and they markedly overlapped among these lineages. In other words, blood stages of the lineages pSGS1 and pGRW4 were indistinguishable from each other during their development in canary and other avian hosts (see Additional file 1: Figure S1, Additional file 2: Figure S2, Additional file 3: Figure S3, Additional file 4: Figure S4).

### Exo-erythrocytic development

Exo-erythrocytic stages were readily visible in a positive control, assuring that the ISH protocol worked. Microscopic examination of the histological sections stained with H&E and the same organ sections treated for ISH did not reveal tissue stages of the parasite lineage pPHCOL01.

### Sporogonic development

Development of ookinetes, oocysts and sporozoites was not observed in exposed *Culex quinquefasciatus* mosquitoes. Only eleven mosquitoes were exposed to pPHCOL01 lineage. Because parasitaemia was barely detectable at sub-microscopic levels in all exposed experimental birds, we were unable to repeat mosquito-infection experiments.

## Discussion

### Relationship of pPHCOL01 to other lineages of *Plasmodium relictum*

This study demonstrate that the new rare lineage pPHCOL01 can be linked to *P. relictum* on both morphological and molecular grounds and provide new data about specificity and development of this infection in experimentally infected avian hosts. This is the first study to compare morphology of blood stages of different lineages of *P. relictum* using the same methodology. Parasites of all examined lineages are typical representatives of sub-genus *Haemamoeba*, whose inclusive species produce large erythrocytic meronts and gametocytes, both of which markedly influence host cell nuclear position (Figs. 2, 3). Morphological forms of blood stages of the parasite lineage pPHCOL01 found in Common chiffchaff, canary and European goldfinch were indistinguishable morphologically. Extensive comparison of blood stages of other *P. relictum* lineages gave the same results (Figs. 2, 3; Additional file 1: Figure S1, Additional file 2: Figure S2, Additional file 3: Figure S3, Additional file 4: Figure S4). Morphological characters, which might be used for distinguishing different lineages of *P. relictum,* were not found because of marked variability of these features during each single infection in all parasite lineages. These data are in accordance with former morphological observations on blood stages of the lineages pSGS1, pGRW4 and pLZFUS01 accessed during experimental exposure of different avian hosts [28–30]. The lineages pSGS1, pGRW4, pGRW11, pLZFUS01, and pPHCOLL01 of *P. relictum* belong to the same *P. relictum* morphotype. Interestingly, the same is true for sporogonic stages of the lineages pSGS1, pGRW11 and pGRW4, which complete development in *Culex pipiens* forma *molestus* mosquitoes synchronously and produce morphologically indistinguishable ookinetes, oocysts and sporozoites at same conditions [32, 33, 46].

None of the bird species that were experimentally infected with lineage pPHCOL01 was good host for investigating dynamics of parasitaemia, and they cannot be recommended for experimental research aimed at studying blood stage infections. Canaries were susceptible, but parasitaemia was transient and light. Zebra finches and one Budgerigar were resistant. Interestingly, the available field observations indicate that the latter two avian species are likely resistant to other *P. relictum* lineages as well. Zebra finches and Budgerigars have never been reported as host of *Plasmodium* parasites by microscopic examination of blood films (this method provides opportunities to visualize blood stages), and probably might resist or tolerate many species of malaria parasites [7, 16, 17]. It is worth noting that Baron et al. [47] reported the lineage pGRW4 in New Zealand budgerigars, indicating that these birds were exposed naturally, but provided no information about whether this parasite completed its life cycle and produced erythrocytic infection in this host species. Development of this parasite might be abortive in Budgerigars, as is the case in many haemosporidian infections [48]. Thus, both Zebra finches and Budgerigars might be excellent model hosts for better understanding mechanisms of innate resistance during avian malaria.

### Biological variation within *Plasmodium relictum*

Molecular techniques that amplify parasite *cytb* genes provide new opportunities to readily distinguish genetically different isolates of *P. relictum* and to identify infections caused by these parasites in avian hosts. This was impossible during the pre-molecular era of malaria research. Numerous molecular studies reported *P. relictum* in naturally infected birds [19, 23, 24, 34, 49], resulting in a solid body of information about the occurrence of these parasite lineages in various avian hosts and ecosystems all over the world (Table 2). However, comparative research on development and virulence of *P. relictum* lineages in different avian hosts and vectors has lagged behind and remains uncommon. This missing information is an obstacle to developing a better understanding of the biological properties of infections caused by different *P. relictum* lineages, limits the ability to predict disease outbreaks, and makes it more difficult to develop adequate steps for improving bird health and conservation.

**Table 2:** Polymerase chain reaction-based reports of *Plasmodium relictum* lineages in avian hosts

Lineage code	Record			
	Zoogeographic region^a^	Bird order	Bird family and no. of positive bird species	Total no. of positive bird species
pSGS1	1, 2, 4, 5, 6	Anseriformes	*Anatidae* (2)^b^	115
		Charadriiformes	*Laridae* (3)	
			*Recurvirostridae* (1)	
			*Scolopacidae* (1)	
		Ciconiiformes	*Ardeidae* (2)	
		Columbiformes	*Columbidae* (1)	
		Galliformes	*Phasianidae* (4)	
		Gruiformes	*Gruidae* (1)	
		Passeriformes	*Acrocephalidae* (6)	
			*Alaudidae* (1)	
			*Certhiidae* (1)	
			*Corvidae* (5)	
			*Emberizidae* (8)	
			*Estrildidae* (1)	
			*Fringillidae* (10)	
			*Furnariidae* (1)	
			*Hirundinidae* (1)	
			*Laniidae* (1)	
			*Motacillidae* (1)	
			*Muscicapidae* (15)	
			*Paridae* (9)	
			*Passeridae* (7)	
			*Passerellidae* (1)	
			*Ploceidae* (4)	
			*Prunellidae* (1)	
			*Pycnonotidae* (3)	
			*Scotocercidae* (1)	
			*Sittidae* (1)	
			*Sturnidae* (2)	
			*Sylviidae* (8)	
			*Thaupidae* (1)	
			*Troglodytidae* (2)	
			*Turdidae* (2)	
			*Tyrannidae* (2)	
		Procellariiformes	*Procellariidae* (1)	
		Sphenisciformes	*Spheniscidae* (1)	
		Strigiformes	*Strigidae* (1)	
		Trochiliformes	*Trochilidae* (2)	
pGRW11	1, 2, 6	Charadriiformes	*Scolopacidae* (1)	41
		Galliformes	*Phasianidae* (2)	
		Passeriformes	*Acrocephalidae* (3)	
			*Alaudidae* (1)	
			*Cettiidae* (1)	
			*Corvidae* (3)	
			*Emberizidae* (1)	
			*Fringillidae* (3)	
			*Hirundinidae* (2)	
			*Laniidae* (1)	
			*Muscicapidae* (6)	
			*Paridae* (4)	
			*Passeridae* (3)	
			*Pycnonotidae* (1)	
			*Sylviidae* (8)	
			*Troglodytidae* (1)	
pGRW4	1, 2, 3, 4, 5, 6, 7	Ciconiiformes	*Ardeidae* (1)	72
		Passeriformes	*Acrocephalidae* (10)	
			*Bernieridae* (2)	
			*Cisticolidae* (2)	
			*Estrildidae* (4)	
			*Fringillidae* (6)	
			*Hirundinidae* (3)	
			*Locustellidae* (2)	
			*Mitidae* (2)	
			*Muscicapidae* (10)	
			*Nectariniidae* (4)	
			*Notiomystidae* (1)	
			*Paridae* (2)	
			*Passeridae* (2)	
			*Philepittidae* (1)	
			*Ploceidae* (6)	
			*Promeropidae* (1)	
			*Pycnonotidae* (1)	
			*Sylviidae* (1)	
			*Thraupidae* (1)	
			*Timaliidae* (1)	
			*Vangidae* (1)	
			*Zosteropidae* (7)	
		Psittaciformes	*Psittacidae* (1)	
pLZFUS01	1, 2, 3, 5	Passeriformes	*Laniidae* (3)	6
			*Parulidae* (1)	
			*Ploceidae* (1)	
			*Pycnonotidae* (1)	
pPHYCOL01	1	Passeriformes	*Phylloscopidae* (1)	1

Modified from MalAvi database (http://www.iucnredlist.org/details/103843725/0)

^a^Zoogeographic regions: 1—Palaearctic, 2—Afrotropic, 3—Nearctic, 4—Neotropic, 5—Indo-Malay, 6—Australasian, 7—Oceanic (borders of the regions were considered according to http://users.tamuk.edu/kfjab02/Biology/Mammalogy/mammalogy_zoogeography.htm)

^b^Number of species is given in parenthesis

Experimental research is essential for better understanding the biology of malaria parasites [5, 8, 11, 33, 50–55]. Controlled experimental studies with *P. relictum* are relatively easy to design due to availability of laboratory-friendly experimental vertebrate hosts (canaries and some species of other common birds), laboratory-colonized susceptible mosquitoes (species of the *Culex pipiens* complex) and worldwide high prevalence in many wild bird species (donors of natural infections). This makes *P. relictum* a convenient and even unique model organism to approach numerous questions about mechanisms of host-parasite interactions, including the immunological aspects during malaria infections [56–58], the ecology and evolution of host-parasite associations [25, 59–63], the host adaptations to tolerate malaria infections [10, 31, 47, 64, 65], patterns of mosquito transmission [32, 46, 53, 66–68] and many other questions.

Unfortunately, experimental information about different lineages of *P. relictum* is still limited, but available data indicate that different lineages and even different isolates of the same lineage might differ remarkably in their ability to develop in different avian hosts and in other biological properties [11]. Brief review what is known about this biological variation is given in the following sections.

### Pathology

The pathology of known lineages of *P. relictum* is highly variable in host species or incompletely known. For example, the same *P. relictum* lineage might cause severe disease in one species of avian host, but other bird species might be tolerant or even resistant [5, 8, 50, 69]. Experimental observations show that the same isolate of pSGS1 behave markedly differently in different species of birds, with the susceptibility ranging from complete resistance to light subclinical (< 0.1%) and high (> 10%) parasitaemia [8, 69]. The variation in parasitaemia dynamics and maximum intensity are often great in different individuals of the same bird species infected with pSGS1 parasite [55]. Similarly, the susceptibility of same bird species to different isolates of the same *P. relictum* lineage also might be markedly different. For example, Hawaiian isolates of pGRW4 readily infect canaries, with maximum parasitaemia ranging from light (about 0.1%) to high (up to 30% and greater) reported in birds exposed by inoculation of infected blood ([70], CTA, pers comm.). However, this bird species was either resistant or had mainly light (< 0.1%) and transient parasitaemia, which rapidly turned to chronic or even latent stages of infection after exposure to European isolates of the same parasite lineage by the same mode of infection ([11], GV, unpublished observation).

It remains unclear why different geographical isolates of the same lineage of *P. relictum* (pGRW4) behave so differently in the same species of birds. The differences between different geographic isolates of *P. relictum* lineages might be due to different clonal intra-lineage genetic diversity, which is great in Hawaiian strains of the lineage pGRW4, but remains insufficiently documented in European isolates of the same lineage [21, 31]. Marked variation in the susceptibility of same experimental bird species to different parasite lineages provide opportunities to use this host-parasite model system for comparative research aimed at a better understanding of the genetic mechanisms of tolerance and virulence during parasitic infections.

Without question, the lineages pSGS1 and pGRW4 are virulent in birds and can cause marked blood pathology and even mortality in susceptible hosts [5, 8, 11, 29, 50, 69]. The negative effects of *P. relictum* (pSGS1) on bird physiological parameters and behaviour are documented due to delicate experimental studies [54, 55]. Observations of infected, naive birds in zoos and rehabilitation centres provided evidence of the severity of disease caused by these and related parasite lineages in wild birds [71–74]. These studies are the basis of understanding the predictions and conclusions of field observations about negative influence of *P. relictum* on population decline or even extinction, particularly on oceanic islands [63, 75–78]. However, to evaluate the true virulence of a malaria parasite lineage in certain avian host species, experimental and field observations are needed, ideally in each targeting host-parasite system separately.

Even though there are numerous reports of exo-erythrocytic stages of *P. relictum* from the pre-molecular research era [1, 7, 13, 84], information about these stages and associated tissue pathology in avian hosts is still absent for parasites of all lineages of *P. relictum*. This is an obstacle to understanding of the mechanisms of persistence in birds, as well as, the association between tissue merogony and pathogenicity caused by different parasite lineages in different avian hosts. This study shows that exo-erythrocytic stages of *P. relictum* can be difficult to find during chronic infections even in experimentally infected birds with visible parasitaemia. This indicates that large multinuclear tissue stages, which are easy to see under light microscopy [6, 13], might persist for a short time and their development might be markedly dependent on the stage of infection. Application of in situ hybridization methods is promising in the investigation of tissue merogony of haemosporidians [6, 37, 78], but may not be sensitive enough to detect uninuclear hypnozoite-like intracellular stages should they occur in *P. relictum*, as is the case in human *Plasmodium vivax* infection. This suggests application of more sensitive immunofluorescent diagnostic techniques in parallel with traditional histology and in situ hybridization methods in research of exo-erythrocytic development of different lineage parasites [1, 6, 35, 37, 78].

Observation of parasites in blood films and determination of morphological characters of their blood stages remain important not only in identification of haemosporidian species [11, 27, 79], but also for distinguishing competent and abortive haemosporidian infections, which might have different consequences for the bird health. During abortive infections, the parasites might circulate within avian hosts as sporozoites or even undergo partial development within non-erythroid tissues, providing templates for PCR amplification, but the parasite would not be able to complete its life cycle due to an inability to enter red blood cells. This would result in absence of gametocytes and other blood stages in the circulation, but severe disease might occur due to damage of internal organs [48]. In the latter case, a positive PCR signal might be obtained, but parasitaemia would be absent or barely detectable due to difficulties in microscopic detection of remnants of tissue stages in the circulation [80–82]. This highlights the relevance of microscopic detection of blood stages and knowledge about morphological features of haemosporidians in pathology and epidemiological studies when used in parallel with molecular diagnostic tools.

### Pre-patent period and parasitaemia

Longevity of the prepatent period cannot be used for distinguishing infections caused by different *P. relictum* lineages. Duration of the prepatent period following sporozoite-induced infection of different lineages of *P. relictum* remains largely undetermined. Prepatent periods have been observed in the Hawaiian parasite lineages pGRW4 where it was within 4 dpe in Iiwi *Drepanis coccinea* and 8 dpe in Hawaii Amakihi *Chlorodrepanis virens* [5, 50]. The prepatent period was about 5 dpe after sporozoite-induced infection of unknown lineage of *P. relictum* in canaries [83, 84].

This study demonstrated that prepatent period of infection is markedly variable in different bird species and individuals of the same species during blood-induced infection of the lineage pPHCOL01. The prepatent period is often about 1 week after the blood-induced infections of pSGS1, but varies markedly in different species of avian hosts and even individuals of the same species even after the same mode and dose of infection, and it might be as long as several weeks after infected blood-induced exposure, indicating the possibility of parasite persistence in internal organs [7, 8, 13, 69, this study].

In all investigated lineages of *P. relictum*, parasitaemia was asynchronous, with trophozoites, growing and mature meronts as well as gametocytes present in the same blood films at the same time in all species of exposed birds at any stage of parasitaemia [8, 29, 30, 33, 70, this study]. This provides opportunities to design vector research with all lineages at any stage of parasitaemia using susceptible avian hosts as donors of infections to expose mosquitoes, but all work carried out to date with different lineages has failed to demonstrate significant differences.

### Host range

An interesting finding of this study is that canaries may not be suitable experimental hosts for all lineages of *P. relictum* and possibly not even isolates of the same lineage. Information about susceptibility of canaries to lineage pLZFUS01 is absent; further experimental studies are needed. This study indicates that canaries can tolerate the pPHCOL01 infection, during which light transient parasitaemia occurs and signs of illness have not been reported. Canaries are good experimental hosts for the lineages pSGS1, pGRW11 and pGRW4 due to long-lasting parasitaemia (usually, several months before latency, with infected birds maintaining infections for several years, with occurring seasonal relapses).

However, infectivity and patterns of development of different lineages and even different isolates of the same lineage might be different, sometimes significantly in canary [11, 70]. A moderate to high (> 0.1% and greater) long-lasting (several months) parasitaemia usually develops during infections with lineages pSGS1 and pGRW11 in canaries exposed by inoculation of infected blood [22, 32, 46]. The same is true for the parasite lineage pGRW4 during development in canaries, but not for all its isolates. For example, the Hawaiian and European isolates of the lineage pGRW4 develop differently in canaries. Hawaiian pGRW4 isolates develop naturally in canaries when caged birds are exposed in habitats with active natural transmission and can develop high (up to 30% and higher) long-lasting parasitaemia after sub-inoculation of infected blood, although significant individual variation is present ([70], CTA, unpublished data). Attempts to induce a long-lasting parasitaemia (several weeks or longer) and gametocytaemia exceeding 0.01% with European isolates of lineage pGRW4 were either completely unsuccessful (compete resistance was recognized in nine exposed birds) or only partially successful with extremely light transient parasitaemia (few gametocytes reported after examination of 100 microscopic fields at high magnification in four birds) ([11], GV, unpublished data). In other words, the canary is not a good host for experimental studies of erythrocytic infections with the European isolates of the lineage pGRW4, but can be used in experiments with the Hawaiian isolate. Experimental studies with other geographical isolates of *P. relictum* (pGRW4) infection have not been performed. Due to relative resistance of canaries to European isolates of lineage pGRW4, Eurasian siskin *Carduelis spinus* has been used in experiments with this parasite lineage, and this species is an excellent experimental host [33].

### Hybridization and gene flow

The lineages pSGS1, pGRW4, pGRW11, pLZFUS01, and pPHCOL01 of *P. relictum* are closely related based on similarities in *cytb* sequence (Fig. 1) and cannot be distinguished by morphology (Figs. 2, 3, Additional file 1: Figure S1, Additional file 2: Figure S2, Additional file 3: Figure S3, Additional file 4: Figure S4). Do these lineages represent distinct species of the *P. relictum* group or are they different genetic variants of the same morpho-species? Do parasites of these lineages maintain the ability to mate? Does the available information provide opportunities to approach answering these questions? This study and available experimental observations [28–30, 32, 33, 46] show that morphological data both of blood and vector stages cannot help in distinguishing parasites of the lineages pSGS1, pGRW4, pGRW11, pLZFUS01, pPHCOL01, indicating that they might belong to the same *P. relictum* morphotype, but some of them also might represent cryptic species of the *P. relictum* group.

Between-lineage hybridization experiments provide opportunities to obtain direct information about the possibility that different lineages of haemosporidian parasites can mate and exchange genetic information. Sexual processes and between-lineage hybridization of *Haemoproteus* parasites (sister genus to *Plasmodium*) can be readily induced in vitro [7]. These experiments indicate probable development of between-lineage *Haemoproteus* parasite hybrids in vitro, which can be readily distinguished morphologically on ookinete stage, but genetic information is lacking, primarily due to obstacles in accessing nuclear genetic information from single cells [85]. A recent molecular study [86] revealed that *cytb* lineages belonging to *Haemoproteus majoris* have unique alleles in 4 investigated nuclear genes and may represent cryptic species. These lineages of *Haemoproteus majoris* are closely related and differ by only 1–6 substitutions over the 479 bp of sequenced *cytb* gene (0.2–1.3% difference). By contrast, an experimental observation in vivo [22] has demonstrated that parasites of the closely related lineages pSGS1 and pGRW11 can mate in mosquitoes *Culex pipiens* forma *molestus* and produce hybrid oocysts. Genetic differences between these lineages in the *cytb* gene are small (0.2%). According to hybridization experiments [22], the parasites of the lineages pSGS1 and pGRW11 are different variants of the same species, but information about hybridization of other lineages of *P. relictum* and other avian haemosporidian parasites is absent.

It is worth noting that partial sequences of merozoite surface protein 1 (msp1) gene were determined in 3 *P. relictum* lineages (pSGS1, pGRW11, pGRW4) in samples collected from different geographic sites using nuclear markers [21]. All three lineages were from markedly randomly sampled birds, with unclear geographical origin of infection. Four different alleles were reported in the lineage pSGS1, and three of them were shared with the lineage pGRW11, indicating possible hybridization. This is in accordance with the available experimental observations [22]. However, five different alleles were revealed in the lineage pGRW4 [21], suggesting the lack of gene flow between parasites of this lineage and the lineages pSGS1 and pGRW11. However, due to the markedly random sampling (many lineage isolates came from different species of African migrants with unclear geographical origin of infection), it is difficult to rule out that the reported genetic difference might reflect strain varieties, but not species differences. Additionally, due to common co-infections of malaria parasites in naturally infected hosts and possible selective amplification of different lineages using general primers [87], it is possible that some samples contained co-infections of different lineages. Because of this, the possibility to create between-lineage nuclear gene artefacts cannot be ruled out as well. In other words, the quality of the haemosporidian sequences should be carefully considered if samples from wildlife are used [88].

*Plasmodium relictum* is a unique among malaria parasites in regard to the enormous range of its avian hosts and mosquito species involved in its transmission. Therefore, direct in vivo experimental hybridization of different *P. relictum* lineages [22] would be most useful if they involved lineage isolates which are transmitted at the same site by the same mosquito species as this would make experimental studies closer to real epidemiological situations that are observed in wildlife.

### Geographic distribution and prevalence

Data about vertebrate host and geographical distribution of different *P. relictum* lineages are summarized in Table 2. The lineages pLZFUS01, pPHCOL01 of *P. relictum* have been reported occasionally, mainly in birds wintering or resident in tropical countries where transmission occurs [30, this study). The parasite lineage pGRW4 has both broad host and worldwide geographical distribution, but is rare in Europe [2, 11, 21, 33]. The lineage pSGS1 and pGRW11 are also broadly distributed, but neither has been reported in several extensive studies in the mainland Americas [2, 21, 89–91]. However, Marzal et al. [3] found *P. relictum* (pSGS1) in 8 native bird species belonging to two orders in Peru, and Quillfeldt et al. [92] reported this parasite in seabirds on Falkland Islands, indicating presence of transmission, at least in South America.

The reported differences in geographical distribution of the lineages pSGS1 and pGRW11 on the one hand, and GRW4 on the other hand are difficult to explain bearing in mind the enormously broad range of their susceptible avian hosts (Table 2) and mosquito vectors, such as the globally distributed *Culex pipiens, Culex quinquefasciatus* and other mosquito species of the *Culex pipiens* complex, which are of global distribution [93–95]. It is worth noting that recent experimental studies have demonstrated complete sporogony of the pGRW4 parasites from European birds, in cosmopolitan *Culex pipiens* forma *molestus* mosquitoes at relatively low temperatures. This indicates that there are no obstacles preventing transmission of this infection in Europe during the warm period of the year [33]. The following explanations of the observed phylogeographic data are worth discussion.

First, the existence of still unclear mechanisms of geographically related limitations in transmission of the parasite lineages pSGS1 and pGRW4 cannot be ruled out. However, the observed results in the phylogeography of these parasites might also originate, at least in part, from bias in DNA amplification of different lineages during co-infections while using general primers [87]. Failure in detection of mixed infections of *Plasmodium* parasites have often been reported [41, 87, 96–98], but have not been investigated among *P. relictum* lineages. In other words, a sensitive issue is that the majority of available studies on *P. relictum* used only general primers for haemosporidian parasite DNA amplification. Such primers are selective and often do not indicate the presence of co-infection of parasites of different lineages [87]. Parasite lineage-specific primers have not been applied in phylogeographic studies of *P. relictum* lineages pSGS1 and pGRW4 and others so far. It remains unclear whether some *P. relictum* lineages are preferably amplified over others, particularly in cases of co-infections of different lineages. Relatively simple experimental studies using the protocol by Bernotienė et al. [87] might be helpful in answering this question. Co-infections of malaria parasites are common and even predominate in some bird populations [87, 96, 97]. This information is essential for better understanding of true distribution of *P. relictum* lineages both by hosts and geographically. Application of specific primers might contribute to better understanding patterns of geographical distribution of these invasive bird infections.

Second, parasite prevalence data depend on both force of infection and the longevity of infection. If local transmission is occurring, the low prevalence of GRW4 infection in European bird populations might be a result of (1) mortality of some European birds due to this infection, as is the case with some endemic Hawaiian birds [1]; (2) resistance and ability of some bird species to tolerate the pGRW4 malaria infection [11]; or, (3) a combination of these two factors. Naive Hawaiian and New Zealand endemic birds suffer mortality from infection with *P. relictum* pGRW4 [5, 50, 75, 77, 99, 100], but introduced bird species are less susceptible and might tolerate this disease [5, 50, 70]. Little is known about the virulence of the pGRW4 infection in resident European birds and other birds worldwide [33]. Preliminary observations indicate that several European bird species (*Fringilla coelebs, Sylvia atricapilla, Passer domesticus*) can resist pGRW4 strains, which were isolated from African migrating Great read warblers *Acrocephalus arundinaceus* [11]. Further experimental studies and application of lineage specific primers might provide more certain information about distribution of these parasite lineages, their co-existence in the same avian hosts and study sites, and better understanding infections in bird health.

### Vector research

The list of mosquito species, which are susceptible to *P. relictum* includes over 20 species [7, 13], however, information about vectors at parasite lineage levels is insufficient [101]. Widespread *Culex pipiens*, *Culex quinquefasciatus* and *Culex tarsalis* mosquitoes are excellent vectors for pSGS1, pGRW4, pGRW11 [22, 32, 33, 46, 52, 102–105], but data about vectors of the pLZFUS01 and pPHCOLL01 parasites are absent. It is interesting to note that mosquitoes belonging to three genera, *Aedes albopictus, Wyeomyia mitchellii* and *Culex quinquefasciatus,* are susceptible to the pGRW4 parasite, and the sporogony was completed in all these mosquito species, but prevalence varied significantly between species. The latter mosquito is the main vector, but other mosquito species might be involved in transmission as well [106]. However, it worth mentioning that, while sporogony was completed in a small fraction of *Wyeomyia mitchellii*, the authors [106] did express doubt in the viability of aberrant sporozoites in this mosquito species.

*Culex quinquefasciatus* is absent in Lithuania. This insect was used in experiments because the new *P. relictum* lineage (pPHCOL01) was isolated from a bird species wintering in Africa where *Culex quinquefasciatus* is widespread [93, 94]. Sporogony of the parasite lineage pPHCOL01 was not initiated in *Culex quinquefasciatus* probably because the donor bird has light gametocytaemia (single gametocytes were seen in donor canaries during mosquito exposure), and that might have been the main obstacle.

Numerous mosquitoes were incriminated as possible *P. relictum* vectors using microscopic methods, but mainly only oocysts were reported in the majority of the studied insects, and the development of sporozoites were accessed in a few species [101]. This questions the conclusions about true possibility and involvement of mosquitoes belonging to different genera to act as effective vectors of *P. relictum* in wildlife. More delicate studies, including the observation of sporozoites in the salivary gland are needed to reach conclusions about ability of certain mosquito species to act as vectors. It is important to note that even presence of sporozoites of *Plasmodium* parasites in salivary glands does not always guarantee that the insects can transmit infection by bite. For example, sporozoites of *Plasmodium hermani* were reported in mosquito *Wyeomyia vanduzeei*, and these sporozoites were used successfully to induce infection in turkeys by syringe inoculation, but this mosquito was unable to transmit infection by bite [107]. This example calls for more delicate vector studies for better understanding transmission of avian haemosporidians. Determination of vectors is time consuming in wildlife studies where diversity of blood-sucking dipteran insects is high. The PCR-based reports of *P. relictum* lineages in wild-caught dipteran insects markedly speed search for possible vectors by indicating significant links between insects, avian hosts and parasites [103, 104, 108–118], but cannot prove that sporozoites develop and can be transmitted by the PCR-positive insects. The observation of *Plasmodium* spp. sporozoites in salivary glands and the studies of transmission by mosquito bites remain the gold standards for determining vector competence. Combination of molecular diagnostic, experimental procedures and microscopic tools remain essential in haemosporidian vector research [33, 46, 101, 106, 119–121].

## Conclusion

*Plasmodium relictum* is a unique species among the large group of parasites causing malaria due to its cosmopolitan distribution and exceptionally broad range of avian hosts and mosquito vectors. These characteristics make the various *P. relictum* lineages exceptional model organisms for better understanding ecological and genetic mechanisms that make generalist pathogens so successful.

Five lineages of *P. relictum* (pSGS1, pGRW4, pGRW11, pLZFUS01, pPHCOL01) have been identified and partially characterized. Parasites of these lineages are phylogenetically closely related, and they cannot be distinguished using morphological characters of their blood or vector stages. Available data show that the same lineages develop markedly differently in different avian hosts. Remarkably, variation among biological properties (prepatent period, parasitaemia dynamics, blood pathology) between different isolates of the same lineage might be greater than the variation between different lineages during their development in the same species of avian host. This indicates the negligible value of these features for diagnosing specific parasite lineages. Currently, the lineages of *P. relictum* can be readily distinguished mainly through mtDNA sequences.

Malaria caused by *P. relictum* is of particular importance for bird health. Controlled laboratory experimental studies show that the lineages pSGS1 and pGRW4 are virulent in birds and can cause marked blood pathology and even mortality in susceptible hosts. However, the exo-erythrocytic stages and tissue pathology caused by them in avian hosts is unknown for parasites of all lineages of *P. relictum*. This is a prominent obstacle for development of the effective prevention and treatment options for birds.

Certainly, more research is needed on biology of *P. relictum* lineages. The existence of still unclear geographically related limitations in transmission of the most prevalent lineages pSGS1 and pGRW4 has been often suspected in explanation of the restricted distribution of these parasites globally. However, methodological issues in the diagnosis of these parasite lineages remain and limit our ability to study co-infections in broadly distributed lineages of *P. relictum*. The information about frequency of co-infection occurrence in lineages of *P. relictum* is inadequate. Mainly general primers have been applied in PCR-based detection and phylogeographic studies of *P. relictum*, and this method is insufficiently sensitive in determining haemosporidian co-infections. It is predicted that available information about both host and geographical distribution of these lineages might be significantly updated if more sensitive diagnostic tools are applied for distinguishing co-infections of these and other *P. relictum* lineages.

Although closely related lineages of *P. relictum* can hybridize, within-species diversity may also indicate the presence of possible cryptic speciation in the *P. relictum* group. Speciation processes have been insufficiently addressed in experimental parasitology studies mainly because of difficulties in accessing and measuring mate-recognition signals in parasites. By focusing on the extracellular sexual process of oogamy, which can be readily visualized both in vivo and in vitro, and the development of oocysts possessing numerous copies of nuclear genes, experimental hybridization can be readily accessed using haemosporidian parasite lineages [22, 33]. Methodologies of between-lineage hybridization of avian *Plasmodium* parasites as well as sister *Haemoproteus* species have been developed [22, 85]. It is important to gain more information about true range of cryptic speciation in pathogens, particularly due to increasingly frequent outbreaks of zoonotic infections, which appear after host switching, leading to the emergence of new diseases [1, 6, 34, 75]. Such studies would also provide directions on how to approach future taxonomic reconstructions on species levels in the genus *Plasmodium* and other haemosporidians. Phylogenetic analysis based on partial *cytb* sequences placed different lineages of *P. relictum* in a tight cluster. Importantly, parasites of these lineages often occur in sympatry in many cases thus, are convenient model organisms to answer questions about the range of cryptic speciation in wildlife malaria and other related haemosporidian parasites.

## Additional files

**Additional file 1: Figure S1.** Mature erythrocytic meronts of the lineage pGRW4 of *Plasmodium relictum* in Hawaiian (a–t) and European (u–x) isolates during development in naturally infected Apapane *Himatione sanguinea* (a–h) and experimentally infected domestic canary *Serinus canaria* (i–x). Note that the size and shape of mature meronts, number of nuclei in them, influence of the meronts on host cells are markedly variable and overlap in both isolates. Meronts of both isolates cannot be distinguished by morphological characters and patterns of their influence on host cells during their development in the same and different avian hosts. Furthermore, meronts of the lineages pGRW4 cannot be distinguished from meronts of the lineage pSGS1 (see Additional file 2: Figure S2). Arrowheads—pigment granules. Giemsa-stained thin blood films. Scale bar = 10 μm.

**Additional file 2: Figure S2.** Mature erythrocytic meronts of the lineage pSGS1 of *Plasmodium relictum* in European isolate during development in experimentally infected Eurasian siskin *Carduelis spinus* (a–h) and domestic canary *Serinus canaria* (i–t). Note that size and shape of mature meronts, number of nuclei in them, influence of meronts on host cells are markedly variable. Meronts of this parasite lineage cannot be distinguished by morphological characters and patterns of their influence on host cells during their development in different avian hosts. Furthermore, meronts of the lineages pSGS1 cannot be distinguished from meronts of the lineage pGRW4 (see Additional file 1: Figure S1). Arrowheads—pigment granules. Giemsa-stained thin blood films. Scale bar = 10 μm.

**Additional file 3: Figure S3.** Mature macrogametocytes (a–o) and microgametocytes (p–x) of the lineage pGRW4 of *Plasmodium relictum* in Hawaiian (a–j, p–v) and European (k–o, w, x) isolates during development in naturally infected Apapane *Himatione sanguinea* (a–f, p–s) and experimentally infected domestic canary *Serinus canaria* (g–j, k–o, t–x). Note that size and shape of mature gametocytes, number and position of pigment granules, morphology of parasite nuclei and influence of gametocytes on host cells are markedly variable and overlap in both isolates. Gametocytes of both isolates cannot be distinguished by morphological characters and patterns of their influence on host cells during their development in the same and different avian hosts. Furthermore, mature gametocytes of the lineage pGRW4 cannot be distinguished from mature gametocytes of the lineage pSGS1 (see Additional file 4: Figure S4). Long arrows—parasite nuclei. Short arrow—vacuole. Arrowheads—pigment granules. Triangle wide arrow—nucleolus. Giemsa-stained thin blood films. Scale bar = 10 μm.

**Additional file 4: Figure S4.** Mature macrogametocytes (a–k) and microgametocytes (l–t) of the lineage pSGS1 of the European isolate of *Plasmodium relictum* during development in experimentally infected Eurasian siskin *Carduelis spinus* (a–g, l–n) and domestic canary *Serinus canaria* (h–k, o–t). Note that size and shape of mature gametocytes, number and position of pigment granules, morphology of parasite nuclei and influence of gametocytes on host cells are markedly variable and overlap during development in different avian hosts. Mature gametocytes of the lineage pSGS1 cannot be distinguished from mature gametocytes of the lineage pGRW4 (see Additional file 3: Figure S3). Long arrows—parasite nuclei. Short arrow—vacuole. Arrowheads—pigment granules. Triangle wide arrows—nucleoli. Giemsa-stained thin blood films. Scale bar = 10 μm.
