# Supplementary figures and images for: Characterization of Plasmodium relictum, a cosmopolitan agent of avian malaria
Source: Malar J. 2018 May 2;17:184. doi: 10.1186/s12936-018-2325-2 (PMC5930738; doi:10.1186/s12936-018-2325-2)

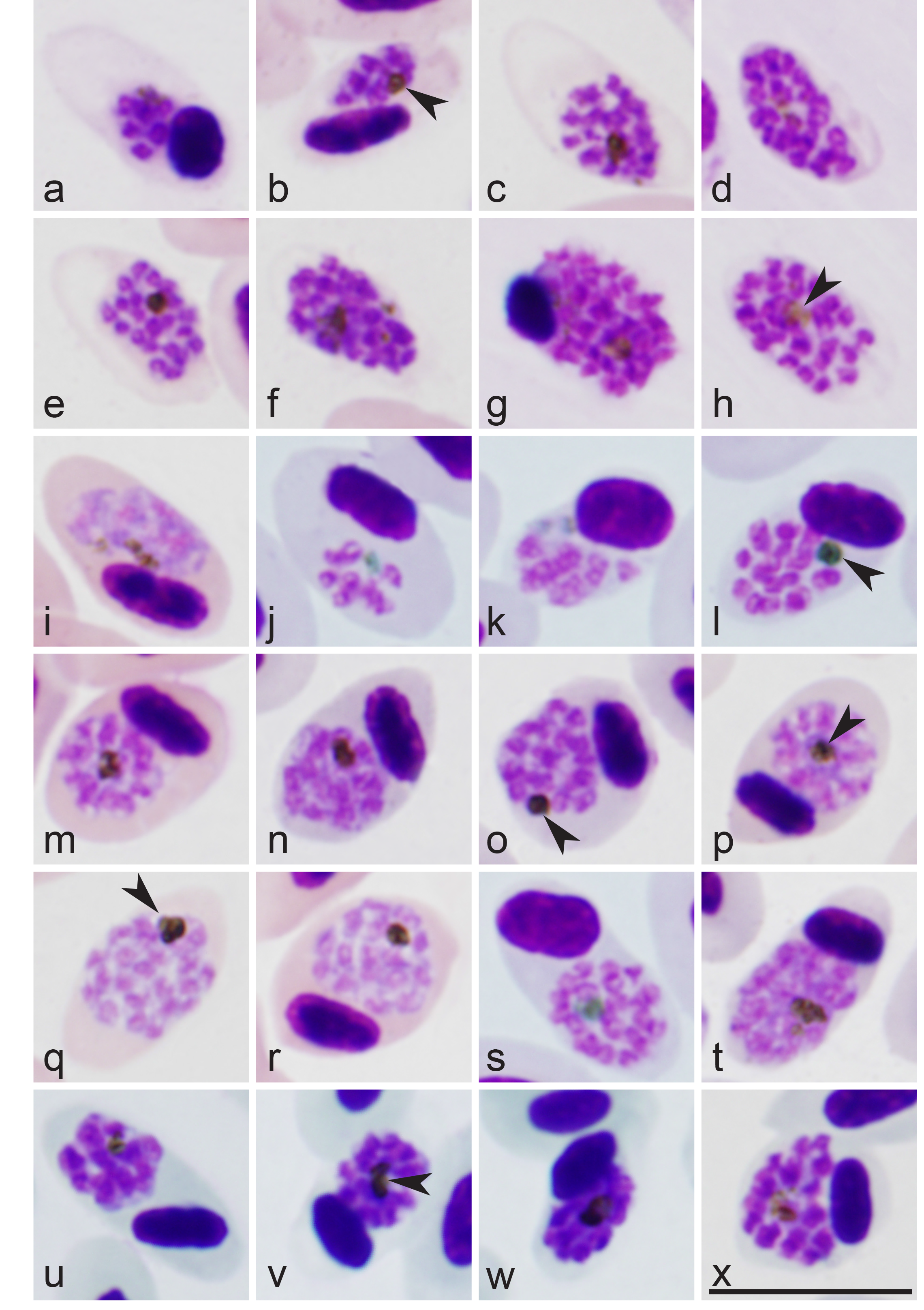

Supplement: Supplementary file 1 — Additional file 1: Figure S1. Mature erythrocytic meronts of the lineage pGRW4 of Plasmodium relictum in Hawaiian (a–t) and European (u–x) isolates during development in naturally infected Apapane Himatione sanguinea (a–h) and experimentally infected domestic canary Serinus canaria (i–x). Note that the size and shape of mature meronts, number of nuclei in them, influence of the meronts on host cells are markedly variable and overlap in both isolates. Meronts of both isolates cannot be distinguished by morphological characters and patterns of their influence on host cells during their development in the same and different avian hosts. Furthermore, meronts of the lineages pGRW4 cannot be distinguished from meronts of the lineage pSGS1 (see Additional file 2: Figure S2). Arrowheads—pigment granules. Giemsa-stained thin blood films. Scale bar = 10 μm. [file 12936_2018_2325_MOESM1_ESM.tif]

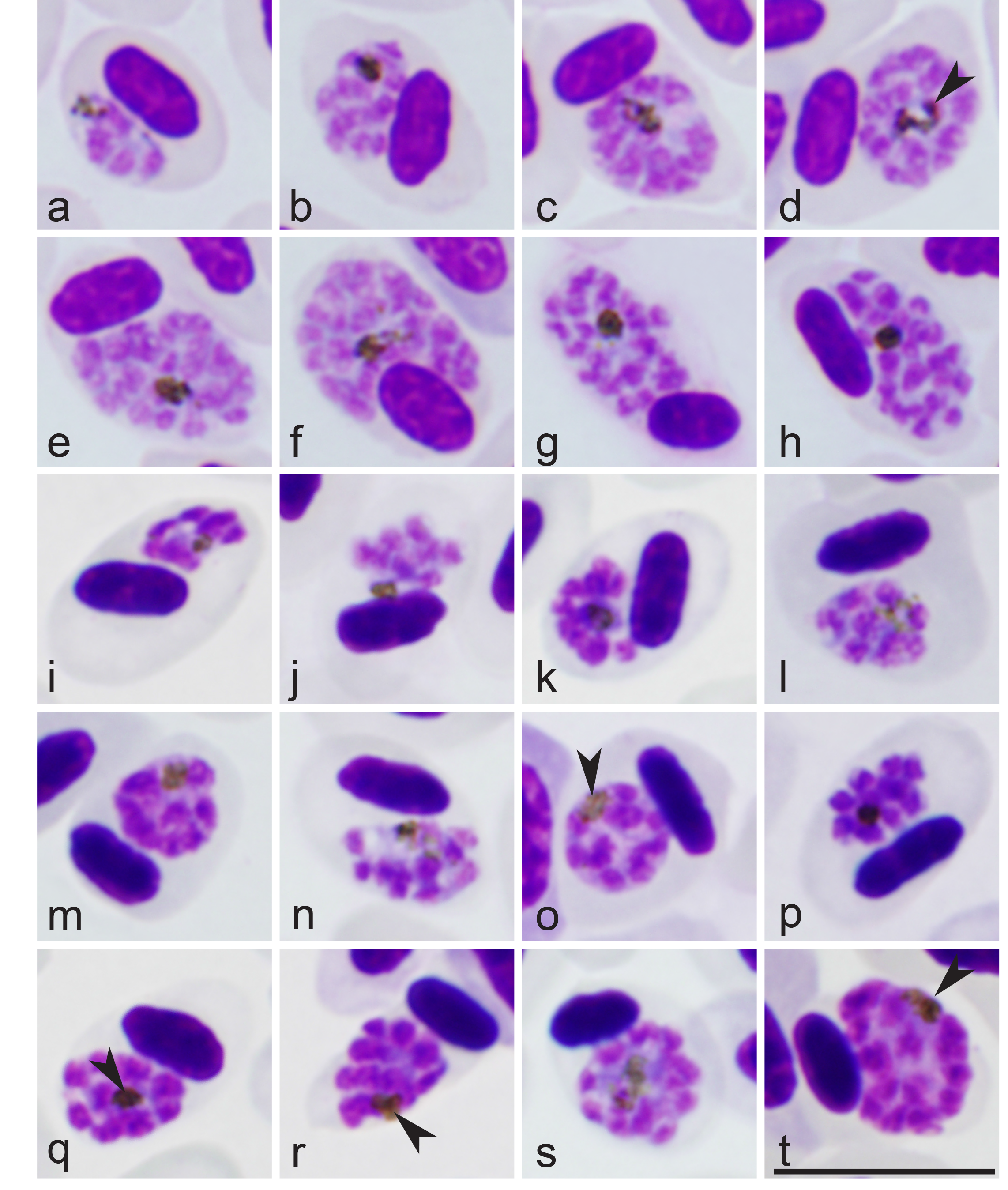

Supplement: Supplementary file 2 — Additional file 2: Figure S2. Mature erythrocytic meronts of the lineage pSGS1 of Plasmodium relictum in European isolate during development in experimentally infected Eurasian siskin Carduelis spinus (a–h) and domestic canary Serinus canaria (i–t). Note that size and shape of mature meronts, number of nuclei in them, influence of meronts on host cells are markedly variable. Meronts of this parasite lineage cannot be distinguished by morphological characters and patterns of their influence on host cells during their development in different avian hosts. Furthermore, meronts of the lineages pSGS1 cannot be distinguished from meronts of the lineage pGRW4 (see Additional file 1: Figure S1). Arrowheads—pigment granules. Giemsa-stained thin blood films. Scale bar = 10 μm. [file 12936_2018_2325_MOESM2_ESM.tif]

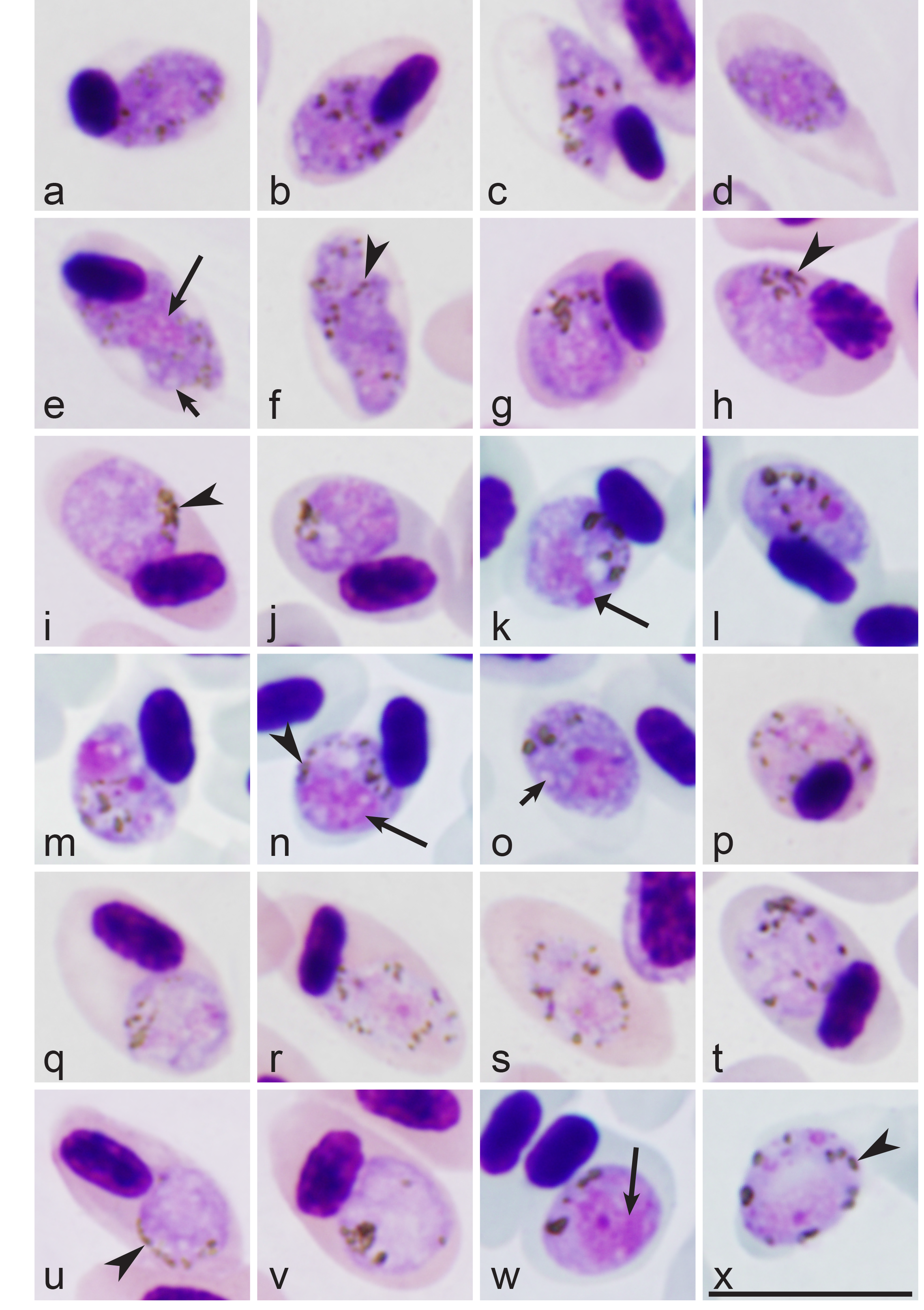

Supplement: Supplementary file 3 — Additional file 3: Figure S3. Mature macrogametocytes (a–o) and microgametocytes (p–x) of the lineage pGRW4 of Plasmodium relictum in Hawaiian (a–j, p–v) and European (k–o, w, x) isolates during development in naturally infected Apapane Himatione sanguinea (a–f, p–s) and experimentally infected domestic canary Serinus canaria (g–j, k–o, t–x). Note that size and shape of mature gametocytes, number and position of pigment granules, morphology of parasite nuclei and influence of gametocytes on host cells are markedly variable and overlap in both isolates. Gametocytes of both isolates cannot be distinguished by morphological characters and patterns of their influence on host cells during their development in the same and different avian hosts. Furthermore, mature gametocytes of the lineage pGRW4 cannot be distinguished from mature gametocytes of the lineage pSGS1 (see Additional file 4: Figure S4). Long arrows—parasite nuclei. Short arrow—vacuole. Arrowheads—pigment granules. Triangle wide arrow—nucleolus. Giemsa-stained thin blood films. Scale bar = 10 μm. [file 12936_2018_2325_MOESM3_ESM.tif]

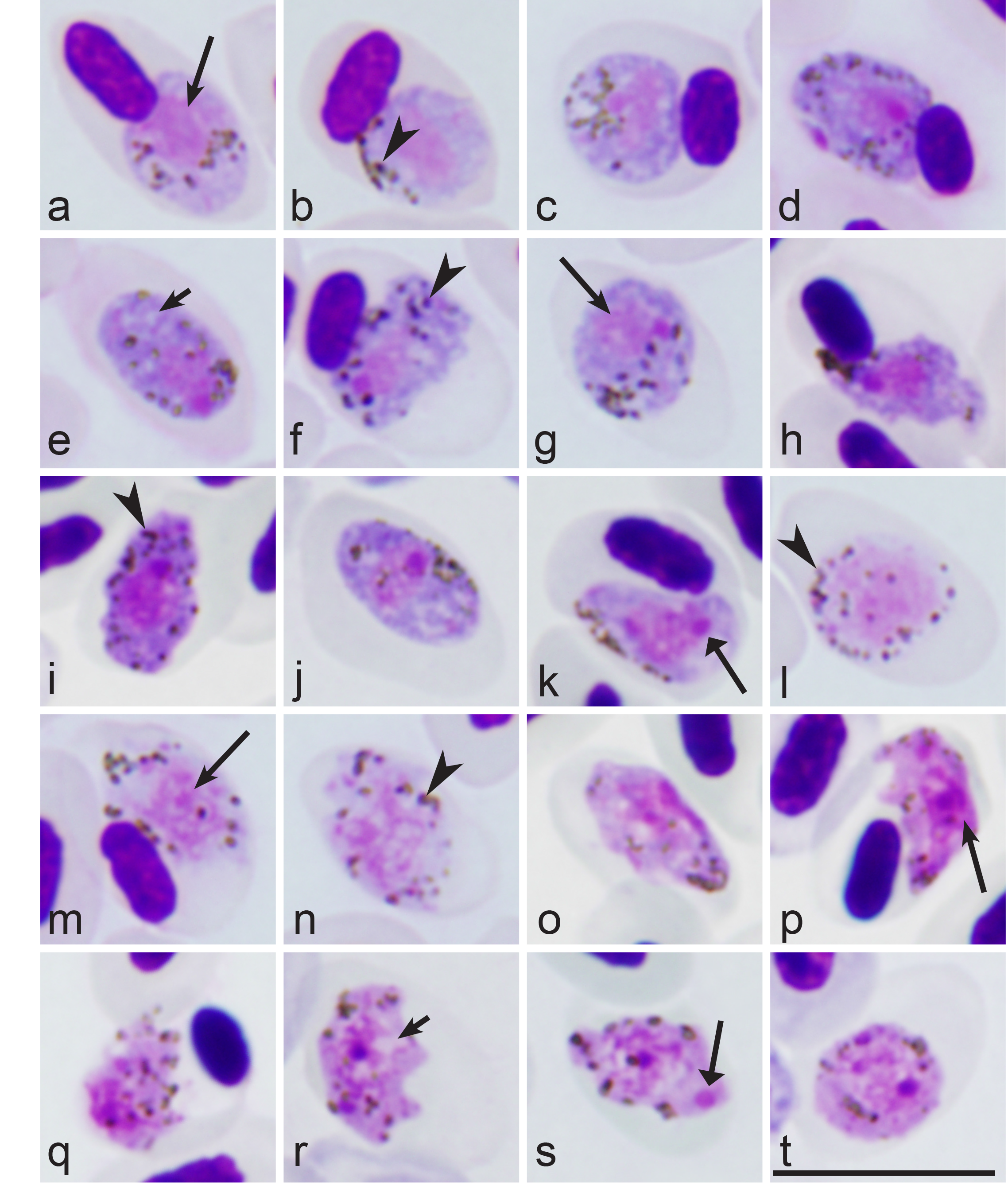

Supplement: Supplementary file 4 — Additional file 4: Figure S4. Mature macrogametocytes (a–k) and microgametocytes (l–t) of the lineage pSGS1 of the European isolate of Plasmodium relictum during development in experimentally infected Eurasian siskin Carduelis spinus (a–g, l–n) and domestic canary Serinus canaria (h–k, o–t). Note that size and shape of mature gametocytes, number and position of pigment granules, morphology of parasite nuclei and influence of gametocytes on host cells are markedly variable and overlap during development in different avian hosts. Mature gametocytes of the lineage pSGS1 cannot be distinguished from mature gametocytes of the lineage pGRW4 (see Additional file 3: Figure S3). Long arrows—parasite nuclei. Short arrow—vacuole. Arrowheads—pigment granules. Triangle wide arrows—nucleoli. Giemsa-stained thin blood films. Scale bar = 10 μm. [file 12936_2018_2325_MOESM4_ESM.tif]
